# Computational Predictions and Microwave Plasma Synthesis of Superhard Boron-Carbon Materials

**DOI:** 10.3390/ma11081279

**Published:** 2018-07-25

**Authors:** Paul A. Baker, Shane A. Catledge, Sumner B. Harris, Kathryn J. Ham, Wei-Chih Chen, Cheng-Chien Chen, Yogesh K. Vohra

**Affiliations:** Department of Physics, University of Alabama at Birmingham (UAB), Birmingham, AL 35294, USA; pabaker@uab.edu (P.A.B.); catledge@uab.edu (S.A.C.); sumner@uab.edu (S.B.H.); katieham@uab.edu (K.J.H.); weichih@uab.edu (W.-C.C.); chencc@uab.edu (C.-C.C.)

**Keywords:** boron-carbon compound, superhard materials, ab initio calculations, chemical vapor deposition

## Abstract

Superhard boron-carbon materials are of prime interest due to their non-oxidizing properties at high temperatures compared to diamond-based materials and their non-reactivity with ferrous metals under extreme conditions. In this work, evolutionary algorithms combined with density functional theory have been utilized to predict stable structures and properties for the boron-carbon system, including the elusive superhard BC_5_ compound. We report on the microwave plasma chemical vapor deposition on a silicon substrate of a series of composite materials containing amorphous boron-doped graphitic carbon, boron-doped diamond, and a cubic hard-phase with a boron-content as high as 7.7 at%. The nanoindentation hardness of these composite materials can be tailored from 8 GPa to as high as 62 GPa depending on the growth conditions. These materials have been characterized by electron microscopy, X-ray photoelectron spectroscopy, Raman spectroscopy, X-ray diffraction, and nanoindentation hardness, and the experimental results are compared with theoretical predictions. Our studies show that a significant amount of boron up to 7.7 at% can be accommodated in the cubic phase of diamond and its phonon modes and mechanical properties can be accurately modeled by theory. This cubic hard-phase can be incorporated into amorphous boron-carbon matrices to yield superhard materials with tunable hardness values.

## 1. Introduction

The first row of elemental solids (C, N, O, and B—jointly referred to as CNOB) form dense covalent solids in three-dimensional (3D) network structures that are extremely hard, have a high-energy density content, and exhibit unique electronic and optical properties. While diamond (hardness of approx. 100 GPa) and cubic-boron nitride (hardness of approx. 45 GPa) have long been established as the cornerstone of a multi-billion dollar abrasives industry, there is considerable scientific and technological interest in novel superhard materials (hardness greater than 40 GPa) based on CNOB. Low pressure/low-temperature synthesis affords the metastable development of unique superhard binary, ternary, and quaternary phases from CNOB precursors that can be quenched to form conformal coatings on a large range of substrates. 

In this paper, we focus on a sub-set of superhard materials based on the boron-carbon system, where the synthesis of superhard BC_5_ material has previously been claimed using the high-pressure high-temperature technique at a pressure of 24 GPa and temperature of about 2200 K [1]. The synthesized BC_5_ material had a measured hardness value of 71 GPa and high thermal stability up to 1900 K [1]. Subsequently, a significant amount of theoretical work has suggested various stable and metastable superhard modifications of boron-carbon systems [2,3,4,5,6,7,8,9]. Our previous study of low-level boron incorporation in diamond by microwave plasma chemical vapor deposition showed a significant change in the plasma gas-phase chemistry and morphology of the diamond films by the introduction of boron in a methane/hydrogen/nitrogen plasma [10]. The focus of this work is on the synthesis of metastable superhard boron-carbon composites from the gas phase using low-temperature microwave plasma chemical vapor deposition. The advantage of microwave plasma chemical vapor deposition (CVD) is its ability for large area synthesis and for overcoming limitations of high-pressure, high-temperature techniques. We have employed gas phase precursors based on hydrogen (H_2_), methane (CH_4_), and diborane (B_2_H_6_) in materials synthesis using a microwave plasma source.

## 2. Materials and Methods

The silicon substrates were obtained from pieces of a <100> oriented, N/Ph doped silicon wafer (University Wafer #1095). These were ultrasonically cleaned with solvents, scratched with diamond powder (2–4 µm particle size) for 30 s on a polishing pad, and then ultrasonically cleaned with DI water and methanol to remove any diamond particles. The B-C films were grown using a 6 kW microwave plasma chemical vapor deposition reactor on a silicon substrate using hydrogen/methane/diborane chemistry. The deposition conditions were: 500 standard cubic centimeters per minute (SCCM), H_2_ as the carrier gas, 22 SCCM CH_4_ as the carbon source, and 0.1–0.45 SCCM B_2_H_6_ as the boron source, and the substrate temperature was carefully controlled in the range of 750–950 °C. The first 45 min of each deposition was performed with only the methane as the precursor to deposit a layer of microcrystalline diamond and the diborane was added to the plasma for the synthesis of high-boron content superhard boron-carbon composites. The deposition conditions are outlined in Table 1 for the four samples described in this manuscript.

The films were analyzed with a Phi Electronics Versaprobe 5000, equipped with a micro-focused Al monochromatic source (λ = 1486.6 eV) and a dual anode conventional X-ray source with a neutralizer. The Mg anode provides X-rays with an energy of 1253.6 eV and the survey spectra were taken with a pass energy of 187.85 eV and a step size of 0.5 eV. Spectra were calibrated such that the C1s peak position was at 284.5 eV. A Panalytical Empyrean X-ray diffractometer was used to obtain diffraction patterns of the films. The optics used were a hybrid monochromator (Kα1 = 1.54059 Å) with a 1/8° divergence slit and a parallel plate collimator on the diffracted beam path with a proportional detector. The films were imaged with a FEI QuantaTM 650 FEG scanning electron microscope. The micro-Raman spectrometer was a Dilor XY with an input laser of 532 nm.

Hardness and Young’s modulus were measured using a NanoIndenter XP with a Berkovich diamond tip with a nominal radius of 50 nm. A common and valid concern is in regard to blunting of the indenter diamond tip when performing hardness measurements on superhard materials. Therefore, in our measurements, we performed calibration of the indenter area function before and after hardness measurements on B-C thin films. A fused silica calibration standard (accepted Young′s modulus of 72 GPa) was tested before and after testing of each CVD-grown sample. All samples, including silica, were indented to a maximum depth of 400 nm. The measured Young′s modulus and hardness values were determined at maximum load and averaged from 10–15 indents (for silica samples), with uncertainty represented as standard deviation of the data. The Young′s modulus of the silica before and after testing all CVD-grown samples was 73.3 ± 0.6 GPa and 74.8 ± 0.9 GPa, respectively. Therefore, the Young′s modulus of the silica calibration standard remained within 4 percent of its average starting value and the indenter tip area function was not modified throughout all tests.

We have performed crystal structure predictions to study stable superhard BC_5_ structures. In principle, one would like to find the stable structure of a compound knowing only its chemical formula by locating the minimum of the Gibbs free energy G = U + PV − TS. It is much more time-consuming to compute the entropy and temperature effects, so quite often only the enthalpy H = U + PV is minimized in practice. The minima of the potential energy surface correspond to different stable and metastable structures, which could potentially be stabilized under different experimental conditions. 

The crystal structure prediction is performed using the USPEX (Universal Structure Predictor: Evolutionary Xtallography) software [11,12,13] based on an evolutionary algorithm developed by Oganov, Glass, Lyakhov, and Zhu. This stochastic method uses concepts such as survival of the fittest and mutation inspired by biological evolution to locate the global minimum of a potential energy surface. The implementation of the algorithm features local optimization, real-space representation, and flexible physically motivated variation operators for highly efficient and accurate structure generation and prediction. For BC_5_, we consider unit cells containing two formula units (12 atoms) and search for the lowest-enthalpy structures in the pressure range of 0–75 GPa with a 5 GPa interval. Among the superhard phases of BC_5_ we found, we then performed an additional structure relaxation calculation at zero pressure to locate the lowest-energy structures, which is a widely used procedure in structure prediction calculation. The ab initio electronic structure calculation is performed using the VASP (Vienna Ab initio Simulation Package) program [14,15]. VASP adopts a plane wave basis set and a pseudopotential method. In our calculations, a plane wave cutoff energy of 600 eV was used, and the projector augmented wave method [16,17] with the PBE/GGA exchange correlation functional [18] was employed. The Γ-centered 22 × 22 × 5 k-point sampling in the Brillouin zone by the Monkhorst-Pack method [19] was used to calculate the total energy summation. For self-consistent and structure relaxation calculations, an energy difference of less than 10^−6^ eV/unit-cell was set for the electronic loop convergence criterion. All structures were relaxed until the forces on each ion were smaller than 10^−3^ eV/Å. The phonon calculations were performed with the density functional perturbation theory method [20] with a 2 × 2 × 2 supercell. An energy difference of less than 10^−8^ eV/unit-cell and forces on each ion of less than 10^−7^ eV/Å were used for the convergence criteria. The resulting interatomic force constants provided inputs for the PHONOPY [21] code to calculate the phonon dispersions and density of states. The lattice parameter calculations for boron-doped cubic diamond were carried out with 16 × 16 × 16 k-point sampling. Single unit cells were used for 0 at%, and 12.5 at% boron concentrations, and a 2 × 2 × 2 supercell was used for 1.563 at%, 3.125 at%, 4.688 at%, 6.25 at%, and 9.375 at% data points. It is noted that the 0 at% case overestimates the lattice constant of pure diamond to 3.5716 Å, which is about a 0.14% error from the accepted value of 3.5667 Å. We also used VASP to compute the elastic modulus tensor, from which the bulk and shear moduli can be calculated, and then the Vickers hardness can be estimated using Chen’s model [22].

## 3. Results

**Figure 1:**
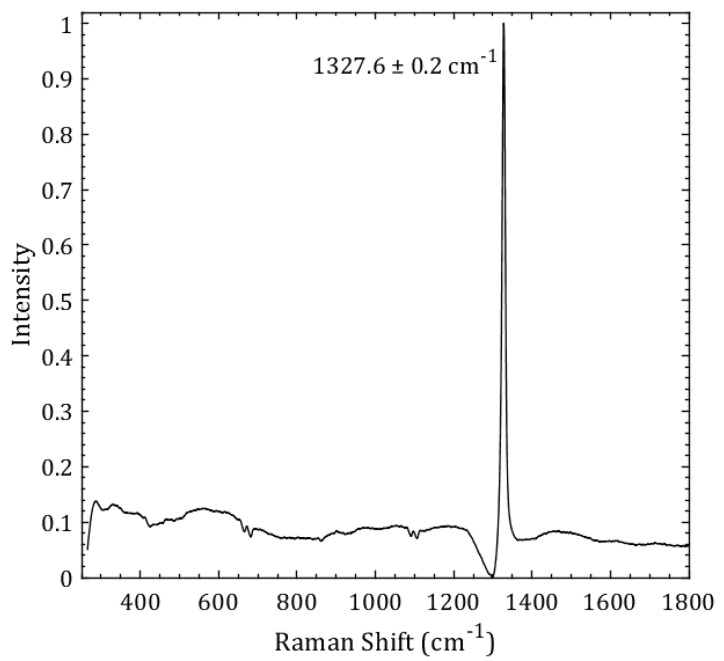
The measured Raman spectrum from a lightly boron-doped diamond (sample LBDD) recorded with the 532 nm laser excitation. A zone-center Raman mode at 1327.6 cm^−1^ is accompanied by weak bands attributed to the Fano effect.

Figure 1 shows the Raman spectrum of a lightly boron-doped diamond film (sample LBDD in Table 1). The Raman spectrum is dominated by a single zone-center mode at 1327.6 cm^−1^ and has a downward shift from a pure cubic diamond peak at 1332.5 cm^−1^. The additional weak features that are observed in Figure 1 are attributed to the Fano effect in boron-doped diamond literature [23,24]. 

**Figure 2:**
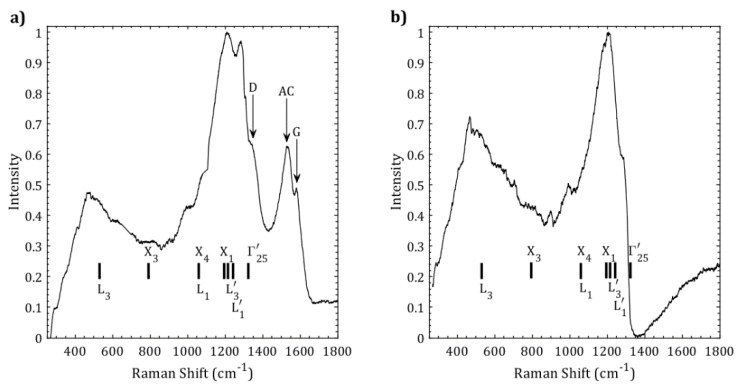
(**a**) Raman spectrum from sample SBCC showing “D” and “G” bands attributed to microcrystalline boron-doped graphite, and amorphous carbon (AC); (**b**) Raman spectrum from sample HBCC showing a hard cubic-phase. The vertical bars show the location of phonon modes in cubic diamond as determined by neutron scattering experiments [25]. The observed Raman modes in HBCC sample are considerably shifted from the cubic diamond positions.

The higher boron-carbon films can be divided into two distinct categories: the ones that are grown at temperatures higher than 900 °C and the ones that are grown below 850 °C. The high temperature films grown above 900 °C contain amorphous boron-doped graphite (soft-phase), while the films grown below 850 °C mostly contain superhard boron-carbon phases. Figure 2a shows the Raman spectrum from the film grown at 925 °C (sample SBCC in Table 1), showing Raman peaks labeled “D” and “G” attributed to amorphous boron-doped graphitic carbon and a peak attributed to amorphous carbon (AC), as well as broad bands attributed to heavily boron-doped diamond. Figure 2b shows the Raman spectrum of a sample grown at 775 °C (sample HBCC), where boron-doped graphitic carbon and amorphous carbon peaks are completely absent. 

In Figure 2, we have also indicated the location of phonon modes observed in cubic diamond by neutron scattering [25]; however, our observed modes are shifted downward in frequency due to the addition of boron in the lattice. The measured Raman frequencies are shown in Table 2 and are compared with the theoretical calculations described later in this paper.

**Figure 3:**
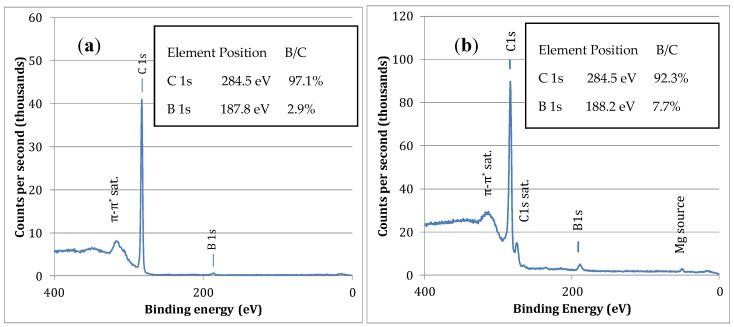
The X-ray Photoelectron Spectroscopy (XPS) analysis of the two boron-carbon films where Raman spectra have been presented in Figure 2. The composition of the film is determined from the intensities of B 1s and C1s emission intensities. (**a**) HBDD sample and (**b**) HBCC sample. The satellite (sat) peaks associated with C1s emission are also labeled.

Figure 3 shows X-ray Photoelectron Spectroscopy (XPS) of the boron-carbon film grown with low and high boron content. XPS is primarily used to quantify the boron-content in the film by comparing the intensity of the B 1s peak to the C 1s peak. In Figure 3a, the boron-carbon film contains 2.9 at% boron and 97.1 at% carbon (sample HBDD in Table 1), and in Figure 3b, the sample contains 7.7 at% boron and 92.3 at% carbon (sample HBCC in Table 1). The value of 2.9 at% boron-doping for HBDD is consistent with the heavily boron-doped samples in the literature, while the 7.7 at% boron is the highest level of boron incorporation in our experiments.

**Figure 4:**
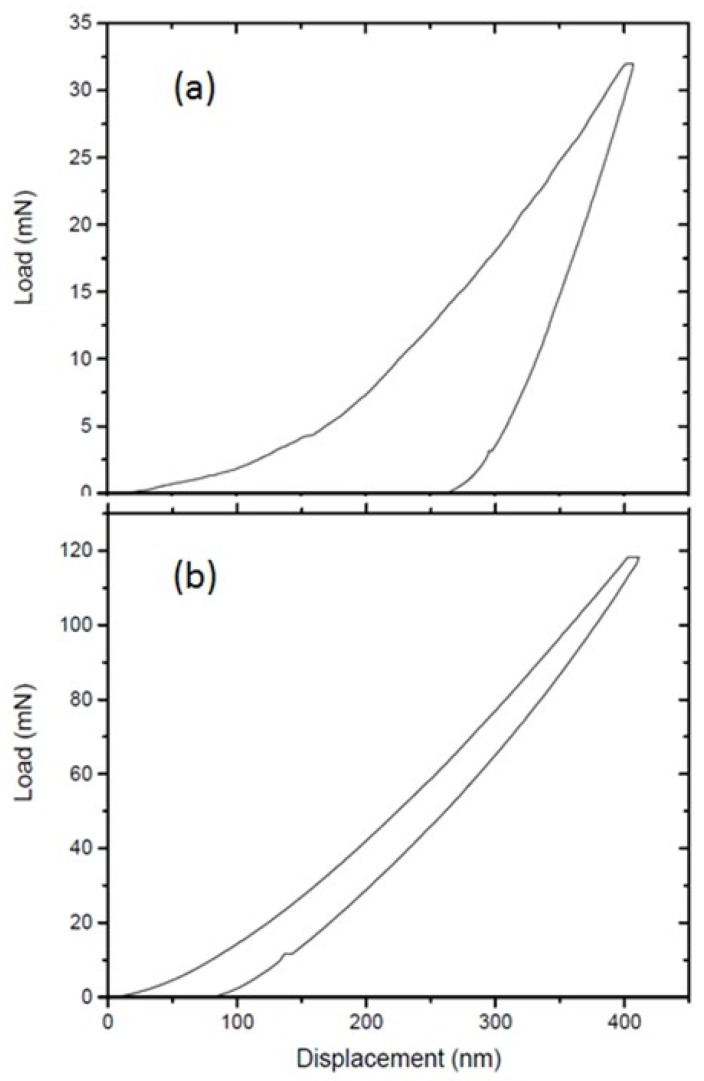
The load displacement curves for the two samples to a depth of 400 nm. (**a**) SBCC sample showing considerable plastic deformation and yielding a hardness value of H = 7.8 GPa and Elastic Modulus of E = 174 GPa based on two such indents and (**b**) HBCC sample showing minimal plastic deformation and yielding a hardness value of H = 61.7 GPa and Elastic Modulus of E = 532 GPa based on seven such indents.

Figure 4 shows the nanoindentation hardness measurements on samples grown at high and low temperatures. Figure 4a shows a load-displacement curve for soft-sample SBCC grown at high temperatures containing microcrystalline boron-doped graphite indented to a depth of 400 nm. The measured hardness (H) and Young′s modulus (E) of the soft-sample are H = 7.8 GPa and E = 174 GPa, respectively. Figure 4b shows a load-displacement curve for hard-sample HBCC grown at low temperatures indented to a depth of 400 nm. The measured hardness and Young′s modulus of the hard-sample are H = 62 GPa and E = 532 GPa, respectively. The relative contribution of elastic and plastic deformation can be calculated from the final unloading depth of the load-displacement curves. The soft sample (SBCC) shows an elastic contribution of 35% and the hard sample (HBCC) shows an elastic contribution of as high as 79%.

**Figure 5:**
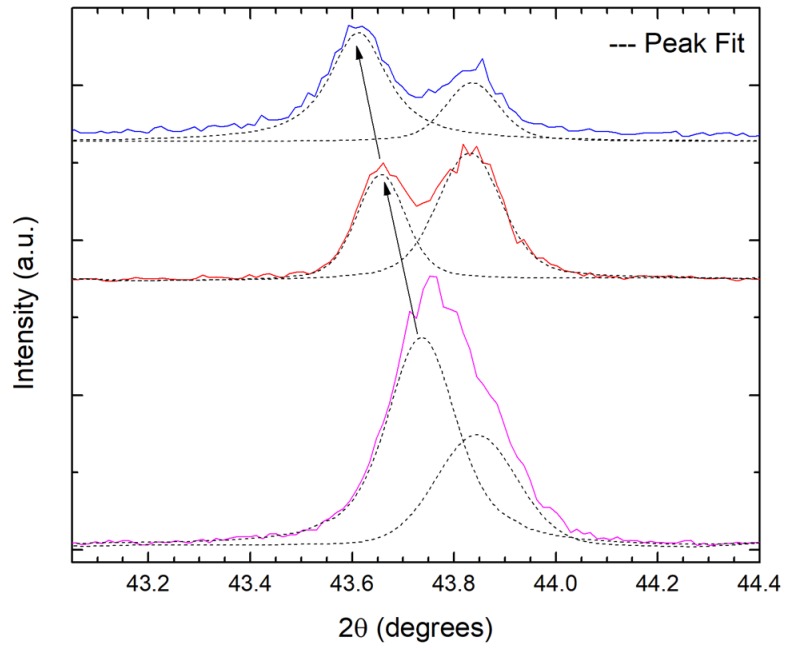
High resolution X-ray diffraction pattern recorded on various B-C films using a hybrid monochromator showing increased splitting of the cubic-diamond (111) diffraction peak with increasing boron-content. The boron-doped diamond peak shift to lower 2θ angles, as indicated by arrows, showing an increase in lattice parameter with increasing boron content.

Figure 5 shows the high-resolution thin-film X-ray diffraction pattern of the B-C samples recorded with monochromatic Cu K_α1_ radiation with a wavelength of λ = 1.54059 Å. The diffraction patterns are indexed to a mixture of two cubic phases, as indicated by the splitting of (111) diffraction peak with increasing boron-content. For the HBCC samples, cubic phases with lattice parameters a_1_ = 3.5743 Å and a_2_ = 3.5917 Å are recorded. The lattice parameter a_1_ corresponds to the underlying cubic-diamond phase and the lattice parameter a_2_ corresponds to the boron-doped hard phase. It should be added that no additional super-lattice X-ray diffraction peaks were observed in addition to the two cubic-phases, as documented above.

**Figure 6:**
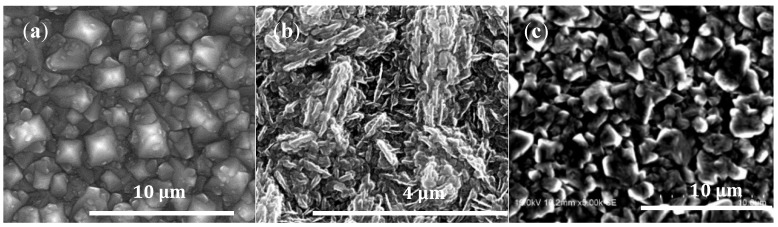
Scanning Electron Micrograph (SEM) of various boron-carbon composites synthesized in this study. (**a**) SEM of the HBDD sample (2.9 at% boron) showing (100) morphology; (**b**) SEM of the SBCC sample containing microcrystalline boron-doped graphite; (**c**) SEM of the HBCC sample.

Figure 6 shows scanning electron micrographs (SEM) of various morphologies observed in our films. Figure 6a shows the typical morphology of an HBDD sample (2.9 at% boron), where (100) cubic growth morphology is apparent in the micrograph. Figure 6b shows a needle-like morphology amorphous boron-doped graphitic carbon sample (sample SBCC) where X-ray diffraction does not show crystalline graphite, confirming the amorphous nature of the deposit. Figure 6c shows the surface morphology of the sample containing 7.7 at% boron (sample HBCC). This morphology shows a less faceted structure than the HBDD sample, possibly indicative of increased crystalline defects (e.g., twinning). 

**Figure 7:**
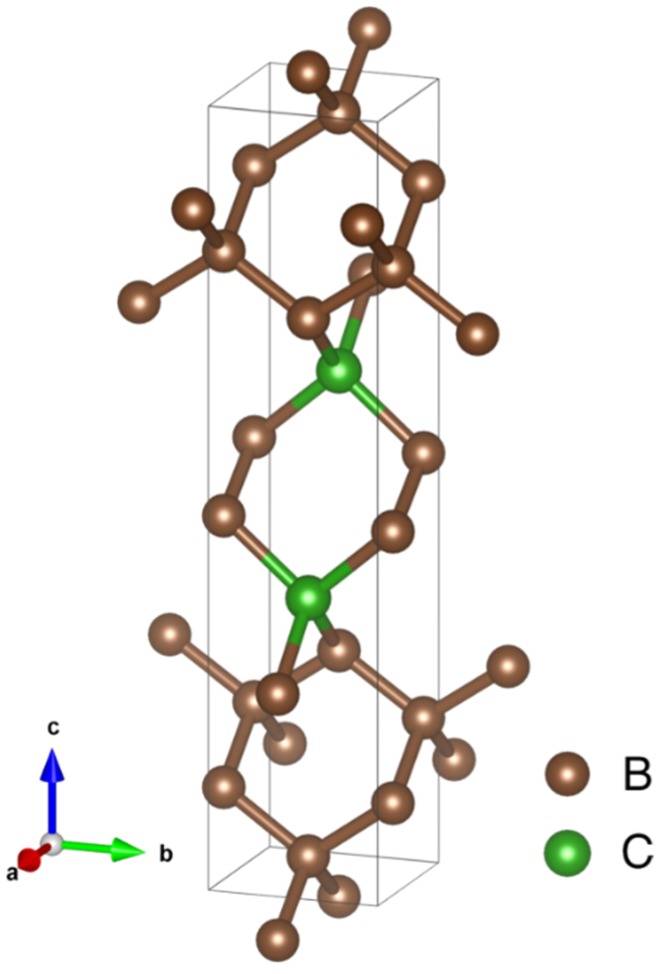
The lowest-energy structure of superhard BC_5_ (containing two formula units) predicted by the evolutionary algorithm as implemented in USPEX [13,14,15]. The unit cell is orthorhombic with Pmma symmetry. The lattice parameters are described in the text. The predicted hardness is 63 GPa.

Among the superhard structures we found, the orthorhombic BC_5_ (containing two formula units) with Pmma symmetry, as shown in Figure 7, has the lowest energy [6]. Its fully relaxed lattice parameters *a*, *b*, and *c* are, respectively, 2.5103, 2.5251, and 11.373 Å. The volume is thereby 6.0076 Å^3^/atom. The bulk and shear moduli are computed to be 378 and 382 GPa, respectively, which yields a Vickers hardness of H = 63 GPa for the structure in Figure 7 using the Chen′s model [22]. In general, similar hardness values are obtained using other hardness models [26,27].

**Figure 8:**
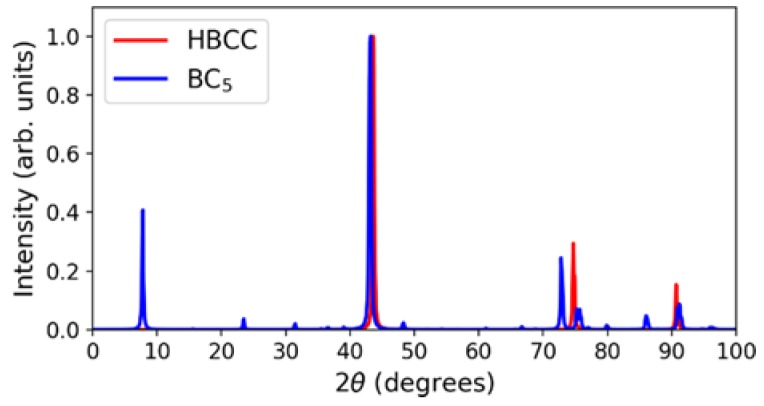
Theoretical X-ray diffraction patterns with 1.5406 wavelength angstrom (Cu K-alpha1) for orthorhombic BC_5_ with Pmma symmetry compared to the experimental X-ray diffraction pattern for the HBCC sample. Each spectrum is normalized by its highest peak intensity.

The corresponding simulated X-ray diffraction pattern is shown in Figure 8 and compared with that of the HBCC sample. We note that there exists another orthorhombic BC_5_, also with Pmma symmetry [6,9]; this structure with direct boron-boron bonding has a slightly higher energy and a predominant X-ray diffraction peak below 10°, which is not observed in our experiments.

**Figure 9:**
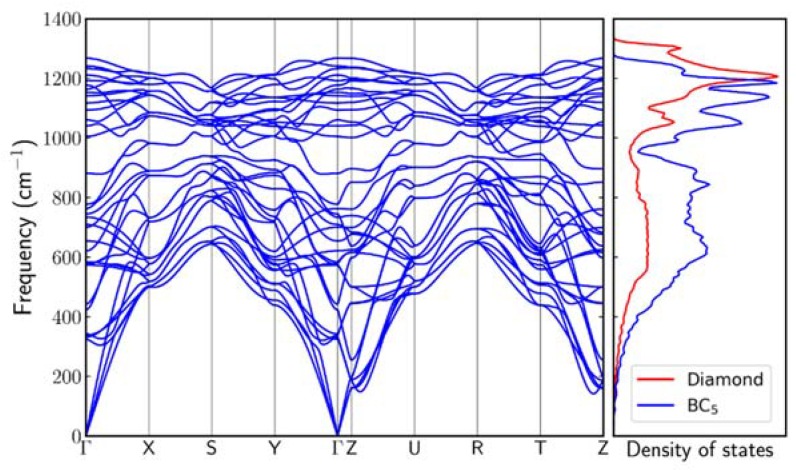
Phonon dispersion and density of states (DOS) of orthorhombic BC_5_ with Pmma symmetry. The phonon DOS of cubic diamond is also shown. Each phonon DOS spectrum is normalized by its peak intensity.

**Figure 10:**
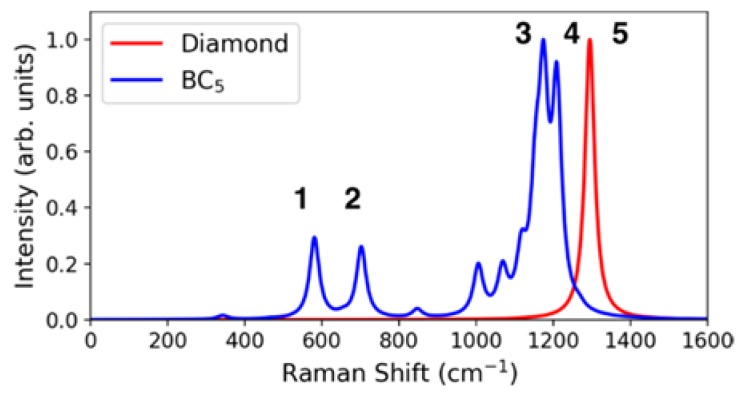
Comparison of the Raman spectrum for the cubic-diamond phase without boron incorporation and the orthorhombic BC_5_ with Pmma symmetry.

We have also performed DFT calculations to investigate the vibrational frequencies of superhard BC_5_ phases at zero pressure. Figure 9 shows the phonon dispersion and phonon density of states (DOS) of the orthorhombic BC_5_ structure in Figure 7. In Figure 9, there is no negative frequency mode identified in the spectra, showing that the theoretical BC_5_ structure in Figure 7 is dynamically stable. The phonon DOS of cubic diamond is also shown for comparison. The existence of boron atoms softens the chemical bonds and shifts the vibrational modes to lower frequencies compared to those in diamond. We note that the pure GGA functional underestimates the vibrational frequencies by a few percent; a more accurate determination of the phonon energies can be obtained using, for example, the B3LYP hybrid functional [28], which is computationally much more expensive. Figure 10 shows the theoretical Raman scattering spectra for diamond and BC_5_ at zero pressure. The single theoretical Raman peak for diamond is located at 1294.7 cm^−1^. For BC_5_, two main theoretical Raman peaks are located at 1209.3 and 1174.7 cm^−1^. Additional low-energy theoretical Raman peaks are located at 702.2 and 508.6 cm^−1^. As shown in Table 2, overall, the theory and experiment are in good agreement.

At a semi-quantitative level, our results show that the main vibrational frequency in BC_5_ softens compared to that in cubic diamond due to slightly elongated atomic bonds, and that broad low-energy peaks emerge due to the presence of boron atoms [9]. These generic features are also observed in other superhard BC_5_ phases and are in good agreement with the experimental Raman measurements, as shown in Figure 2. 

**Figure 11:**
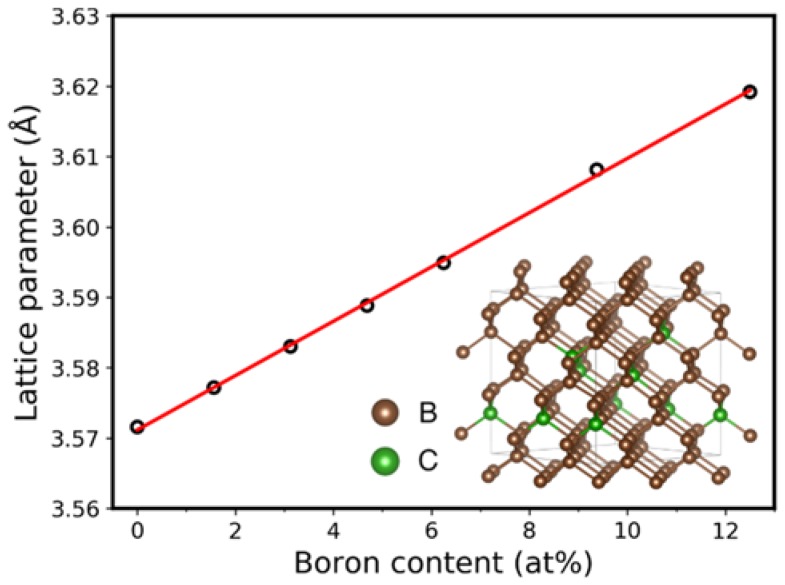
Plot of DFT calculation showing lattice parameters for cubic diamond structure as a function of the boron content. The linear behavior is predicted by Vegard′s Law. The inset structure shows a 2 × 2 × 2 supercell of boron-doped cubic diamond with a boron content of 9.375 at%.

Further DFT calculations were performed to estimate the lattice parameter of the cubic diamond structure as a function of at% boron doping. The calculation predicts that the lattice parameter linearly increases with increasing boron content, in accord with the Vegard′s law [29]. Figure 11 shows the calculated points, as well as a linear fit with equation *y* = 0.003859*x* + 3.5712.

The DFT calculations allow supercells of boron-doped diamond to relax while maintaining a cubic diamond structure when the boron content does not exceed 12.5 at%. The maximum lattice parameter measured by XRD of the thin films is 3.5917 Å. Using this value with the linear fit equation, the calculations predict a boron content of 5.3 at%. It should be added that our measured value of the lattice parameter of 3.5917 Å is similar to 3.589 Å, which is the value reported for the cubic phase of BC_3_ [30], and 3.59 Å, the value reported for the cubic phase of BC_5_ [1]. This indicates that our HBCC film consists of highly doped diamond, containing 5.3 at% boron in a matrix of amorphous boron-doped graphitic carbon which accepts the remaining 7.7 at% boron, as measured by XPS.

## 4. Discussion

Aided by evolutionary algorithm predictions, we have synthesized a novel series of boron-carbon materials using a microwave plasma chemical vapor deposition technique employing hydrogen/methane/diborane gas-phase chemistry. The hardness of the film can be varied from 8 GPa to as high as 62 GPa depending on the growth conditions, thus opening the door for superhard materials synthesis suitable for high temperature operations. We have used DFT calculations to explain our experimental results, achieving overall good theory-experiment agreements on the measured hardness and vibrational spectra recorded by Raman spectroscopy. The theoretically predicted stable orthorhombic structure for stoichiometric BC_5_ (16.7 at% boron) was not observed in our metastable synthesis from the gas-phase. Instead, we document a metastable cubic diamond-like phase all the way to the highest boron concentration of 7.7 at%. Our data analysis also indicates that the lattice parameter of the cubic hard phase synthesized by the microwave plasma method is similar to the hard phases reported in BC_3_ and BC_5_ materials that have been synthesized by high-pressure high-temperature techniques. Low pressure/low-temperature plasma synthesis from the gas phase of the superhard boron-carbon composites offers advantages over high-pressure high-temperature methods in terms of large area deposition on a variety of substrates.

## Figures and Tables

**Table 1:** Growth conditions for the four samples described in this study. The nomenclature of samples is as follows: LBDD = lightly boron-doped diamond, HBDD = heavily boron-doped diamond, SBCC = soft boron-carbon composite, and HBCC = hard boron-carbon composite.

Sample	Growth Temp. (°C)	Microwave Power (W)	Chamber Pressure (Torr)	CH_4_ (SCCM)	B_2_H_6_ (SCCM)	Growth Time h (Diamond/B-C)
LBDD	875	900	58	22	residual	5.5 h/N-A
HBDD	845	850	51	22	0.15	2/2.8
SBCC	925	900	60	22	0.15	0.75/6.3
HBCC	775	850	53	22	0.45	0.77/6.8

**Table 2:** Measured Raman Frequencies of spectra shown in Figure 1 and Figure 2.

Sample	Peak 1 (cm^−1^)	Peak 2 (cm^−1^)	Peak 3 (cm^−1^)	Peak 4 (cm^−1^)	Peak 5 (cm^−1^)	Peak 6 (cm^−1^)	Peak 7 (cm^−1^)	Peak 8 (cm^−1^)
SBCC	463.9 ± 2.0	684.8 ± 17.4	1084.8 ±14.4	1209.6 ± 2.9	1287.1 ± 0.5	1346.1 ± 5.8	1530.0 ± 1.1	1587.3 ± 1.0
HBCC	466.7 ± 2.1	705.8 ± 29.0	1078.5 ± 8.3	1208.2 ± 0.8	1290.9 ± 0.5	—	—	—
LBDD	—	—	—	—	1327.6 ± 0.2	—	—	—
Theory	508.6	702.2	1174.7	1209.3	1294.7	—	—	—
